# Structure Optimization of Polymerase Chain Reaction Devices Under High Flow Rate: A Numerical Study

**DOI:** 10.3390/mi17010021

**Published:** 2025-12-24

**Authors:** Naixiang Zhou, Hao Han, Liwei Fang, Shizhen Li, Li Lei

**Affiliations:** 1 School of Nuclear Science, Energy and Power Engineering, Shandong University, Jinan 250061, China; zhounaixiang@sdu.edu.cn (N.Z.); 202314507@mail.sdu.edu.cn (H.H.); 2 Shandong Huineng Energy Saving Equipment Co., Ltd., Jinan 250308, China; sdhnjn@163.com; 3 Institute of Marine Science and Technology, Shandong University, Qingdao 266237, China; lishizhen@sdu.edu.cn

**Keywords:** polymerase chain reaction, continuous flow, microchannel, temperature uniformity

## Abstract

Polymerase chain reaction (PCR) is vital in biological and medical research, but microfluidic PCR chips often suffer from limited reagent processing capacity and slow thermal response under high flow rates. To address this, we designed three serpentine microfluidic chips with double-sided heaters: a standard serpentine chip (case 1), one with unchamfered channel expansion areas (case 2), and one with chamfered expansions (case 3). Using numerical simulations, we analyzed temperature, velocity, and pressure distributions at flow rates of 75, 125, and 175 μL/min. At 175 μL/min, case 2 showed a 41% higher pressure drop than case 1, but also demonstrated significantly improved thermal performance: the constant-temperature zones were extended by 30 mm, 10 mm, and 30 mm at 95 °C, 72 °C, and 55 °C, respectively; the temperature gradient in expansion zones increased by 1.6 times; and the maximum temperature difference decreased by 80%. Case 2 achieved the best trade-off between thermal performance and flow resistance, making it suitable for high-flow-rate PCR applications.

## 1. Introduction

Polymerase chain reaction (PCR), first invented by Kary Mullis [1], is a molecular bi-ology technology whose principle is similar to the natural replication process of DNA. It realizes rapid amplification and replication of specific DNA fragments through repeated thermal cycle procedures such as high temperature denaturation, low temperature annealing, and warm extension. It is widely used in gene research, food safety, medical diagnosis, and other fields. Microfluidic technology refers to the technology of processing or manipulating trace fluids (10^−9^ to 10^−18^ L) using flow channels with a size of tens to hundreds of microns [2]. Compared with traditional systems, the reaction speed, mass transfer, and heat transfer inside microfluidic chips are improved, making them very suitable for rapid biological analysis such as protein analysis and gene analysis [3]. Various micro-fluidic platforms are designed for applications related to lab-on-a-chip devices [4], which can provide results in fast turnaround times with a small amount of sample reagents compared to laboratory testing.

Microfluidic PCR devices can be divided into two types: continuous flow PCR devices and stationary chamber PCR devices. In a stationary chamber PCR device, the sample is static, and the temperature of the PCR reaction chamber cycles between three different temperatures [5] to achieve the amplification of DNA fragments. In comparison with a stationary chamber PCR device, the microfluidic continuous flow PCR device is a compact alternative that eliminates the need for high-capacity thermal heaters and temperature controllers in conventional devices, and the heating and cooling rates of PCR amplification are limited by the flow rates of PCR mixtures in microchannels [6]. Numerous researchers have employed numerical simulation methods to investigate the thermal performance of PCR chips [7,8,9,10,11]. Nakano et al. [12] developed a new tubular simple reactor for PCR using a thin Teflon capillary tube as a tubing reactor in which the reaction mixture of PCR is driven by a pump at a constant flow rate and the sample is treated with three continuous heating segments for denaturation, annealing, and extension of DNA and primers, with a total reaction time of 12 to 18 min. KOPP et al. [13] developed a continuous flow PCR device in 1998, in which a sample is continuously passed through the channel of a microfluidic chip, repeatedly flowing through three temperature zones to achieve DNA amplification, which is designed to speed up the reaction. For PCR thermal cycling systems, temperature directly affects the amplification process of PCR. Chou et al. [14] designed and fabricated a miniaturized cyclic PCR device in low-temperature ceramics that includes a serpentine channel with different cross-sectional areas within different reactor zones to provide sufficient residence time for denaturation, annealing, and extension reaction to occur, which is smaller, easier to integrate, and reduces consumables compared to standard PCR systems. Kim et al. [15] used polydimethylsiloxane (PDMS)/glass laminate to fabricate continuous flow microchips heated by aluminum blocks to achieve efficient PCR, and investigated the effects of extension time and polyvinylpyrrolidone (PVP)-treated microchannels on PCR efficiency. Mohr et al. [16] built a double-temperature continuous flow PCR device using a serpentine channel, and found that the flow rate had a great influence on the droplet residence time. When the flow rate was higher than the critical flow rate, the sample could not reach the temperature required for DNA amplification. Sun et al. [17] proposed a new continuous flow hybrid PMMA-PC microchip, and they found that the PCR efficiency obtained in the PMMA-Polycarbonate (PC) microchip was higher than that of the PMMA-PMMA microchip, and the hybrid PMMA-PC microchip was more suitable for high-temperature applications. Moschou et al. [18] demonstrated a continuous flow microfluidic PCR device for DNA amplification by PCR with integrated heating elements on commercially available thin polyimide substrates. Tachibana et al. [19] created a new disposable plastic-based microfluidic PCR chip that provides a fast, easy-to-use, and low-cost real-time PCR system with the potential to be used for on-site gene testing. Abed et al. [20] pointed out that the serpentine channel has the inherent advantage of secondary flow structure and is one of the ideal choices for heat transfer enhancement in micro-devices. Madadelahi et al. [21] designed and fabricated a high-throughput two-phase PCR device using a serpentine microchannel together with a spiral structure, and achieved a five-fold performance improvement using diamond nanoparticles-assisted PCR reaction. Chang et al. [22] proposed a simple and efficient bond technique integrating PDMS and printed circuit board (PCB) by using the half-cured PDMS technique. Based on the bond technology, a continuous flow PCR chip was also fabricated for DNA amplification. Zagklavara et al. [23] proposed an optimization method based on computational fluid dynamics for a continuous flow PCR device with a serpentine channel structure. This method can observe the optimization of DNA amplification efficiency and pressure drop while changing the width and height of the microfluidic channel. It is possible to increase the DNA concentration and pressure drop in the PCR cycle by 2.1% and 95.2%, respectively. Mota et al. [24] designed and manufactured a microfluidic device capable of generating a large number of uniform droplets with volumes in the nanoliter range. Hsieh et al. [25] combined PCR with a gold nanoslit-based surface plasmon resonance (SPR) sensor for detecting the DNA sequence of latent membrane protein 1 (LMP1); the device can complete the analysis procedure in about 36 min, compared to 105 min for a conventional machine to obtain a similar signal under the same PCR thermal cycle. Indulakshmi et al. [26] numerically studied the phase changing materials (PCM)-assisted annealing process in the continuous flow PCR system, and developed an unsteady one-dimensional thermal model for sample fluid flow in microfluidic channels containing PCM. The results obtained from this one-dimensional thermal model were compared with two-dimensional computational fluid dynamics simulations performed using a finite volume solver based on the enthalpy-porosity method. The results show that the PCM-laden channels provide excellent passive control of the annealing process in a continuous flow PCR system. Zagklavara et al. [27] proposed a multi-objective optimization method for continuous flow PCR systems. They used a combination of conjugate heat transfer simulation and machine learning to create accurate alternative models of DNA amplification efficiency, total residence time, total substrate volume, and pressure drop for a practical continuous flow PCR device with sigmoid-shape microfluidic channels. The results showed that DNA amplification is closely related to residence time in the extension zone. However, the residence time in denaturation and annealing zones is not related. Chen et al. [28] integrated a Peltier element onto a microfluidic continuous flow PCR chip to adjust the annealing temperature. They used the surface characteristics to examine the protein absorption effect on the surface of hydrophobic PDMS, and the results showed that a 385-bp segment of Coxiella burnetii DNA was successfully amplified in the DNA amplification system. Yang et al. [29] proposed a fabrication method for three-dimensional spiral microreactors using metal molds and PDMS, and analyzed the heat transfer of the microreactor using commercial software. The amplification efficiency was 87% and 85%, respectively.

Despite these advances, a significant challenge remains in achieving rapid and uniform temperature transitions under high flow rates, which is critical for high-throughput PCR applications. Existing designs often suffer from prolonged thermal inlet sections and non-uniform temperature distribution, limiting their efficiency. To address this, we pro-pose a novel serpentine microfluidic chip with integrated channel expansion zones to enhance thermal performance without compromising flow capacity. Original flow channel expansion areas were chamfered to create two different chip structures, considering that the flow channel expansion areas were blocked. Through numerical simulation, this paper compared the fluid temperature distribution, velocity distribution, and pressure loss between two types of serpentine double-sided heating microfluidic chips with flow channel expansion areas and an ordinary serpentine double-sided heating microfluidic chip. Furthermore, the influence of the flow channel expansion areas on fluid flow and heat transfer characteristics within the chips was investigated.

## 2. Numerical Simulation and Verification

### 2.1. Physical Model

**Figure 1:**
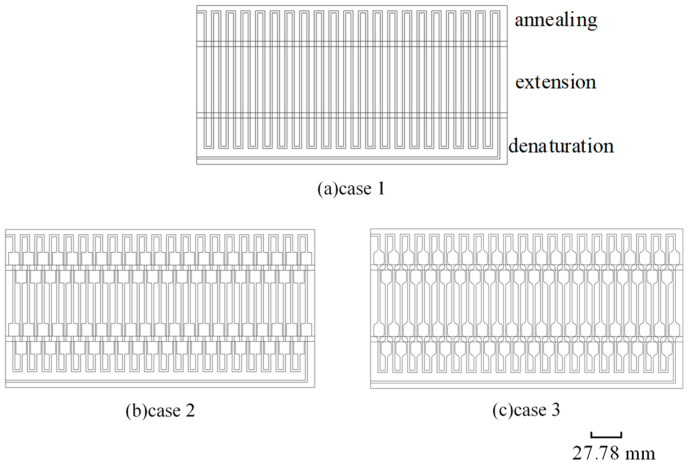
Serpentine double-sided heating microfluidic chips with three different channel structures.

In this numerical simulation, we employed three different three-dimensional structured continuous flow PCR chips with double-sided integrated heaters, as shown in Figure 1. Case 1 is an ordinary serpentine double-sided heating microfluidic chip, case 2 is a serpentine double-sided heating microfluidic chip with unchamfered flow channel expansion areas, and case 3 is a serpentine double-sided heating microfluidic chip with chamfered channel expansion areas. Channels are engraved inside the chip, the total length of the channels is 5831.4 mm, the width is 2 mm, the depth is 1 mm, and 20 DNA amplification cycles can be achieved. There are three heating blocks with different temperatures distributed above and below the chip, and there are intervals between the heating blocks. The temperature of denaturation, extension, and annealing zone are set at 95 °C, 72 °C, and 55 °C respectively, and the ratio of the length of the flow channel in the three areas is 1: 2: 1.

**Figure 2:**
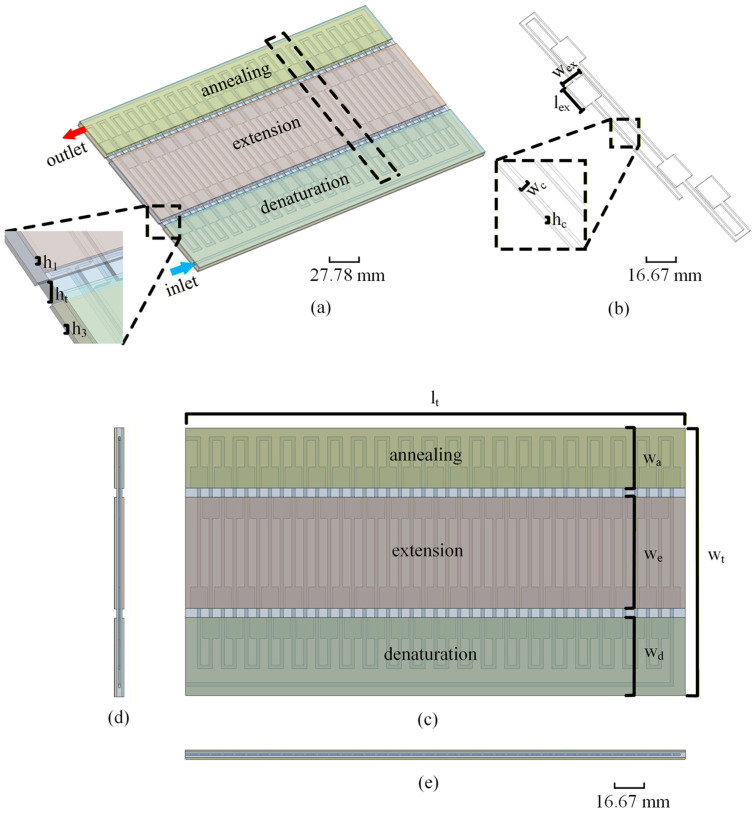
Numerical model of a serpentine double-sided heating microfluidic chip with flow channel expansion areas: (**a**) overall model; (**b**) local flow channel; (**c**) top view; (**d**) left view; (**e**) front view.

Case 1 is an ordinary serpentine double-sided heating microfluidic chip; the flow channel width is unchanged. Case 2 is a serpentine double-sided heating microfluidic chip with unchamfered flow channel expansion areas. Compared with case 1, case 2 is special in that every time the fluid flows from one temperature zone to the next temperature zone, it first needs to pass through an area with a larger channel width, which is called the flow channel expansion area, and then through the ordinary straight flow channel. Considering the possible problem of fluid flow stagnation, case 3 is obtained by chamfering the flow channel extension areas based on case 2. Therefore, case 2 is taken as an example to introduce the numerical model used in this study. Figure 2 shows a schematic diagram of the geometric model adopted in the numerical simulation process. Table 1 shows the geometric parameters of the three structural models.

In this simulation, the chip material is PMMA, the fluid in the flow channel is water, and the heating block material is copper. This study assumes that the physical properties of the working medium are unchanged, and the specific physical parameters are shown in Table 2.

### 2.2. Governing Equations and Boundary Conditions

In this study, ANSYS Fluent 2023R1 software is used to perform simulations under laminar flow conditions. The mass conservation, energy conservation, and momentum conservation equations, which govern fluid flow and heat transfer, are employed to solve the problem. The governing equations [30] adopted for single-phase flow are presented as follows:

Continuity equation:

∇·u = 0(1)

Momentum equation:

(u·∇) ρ_1_ u = −∇P + μ∇^2^ u (2)

Energy equation:

ρ_l_ c_p,l_ (u·∇T) = k_l_ ∇^2^ T (3)

where u, P, and μ represent fluid velocity vector, pressure, and dynamic viscosity, while c_p,l_, T, and k_l_ represent water specific heat, temperature, and water thermal conductivity, respectively.

The simulation process is based on a three-dimensional steady-state numerical simulation using the pressure solver. The coupling equations for pressure and velocity are solved using the Coupled algorithm. The pressure interpolation method employs the Second Order interpolation format, while both the energy and momentum equations are computed using the Second Order Upwind format. The inlet boundary condition uses the mass flow inlet, and the inlet volume flow rate is 75 μL/min, 125 μL/min, and 175 μL/min respectively, and the temperature is 26.85 °C. The outlet boundary condition uses a pressure outlet. The temperatures of heating blocks arranged above and below the chip are 95 °C, 72 °C, and 55 °C, respectively. The contact surface between the heating blocks and the chip uses a constant wall temperature boundary condition. Ideal thermal contact is assumed between the heating blocks and the chip, neglecting contact resistance, as the heaters are integrated into the chip structure.

### 2.3. Grid Independence Verification

**Figure 3:**
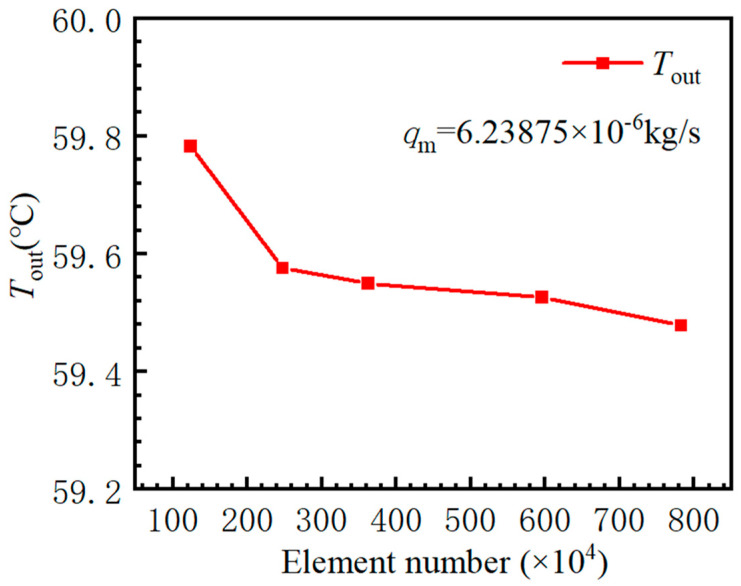
Grid independence verification.

To ensure that the numerical simulation results are independent of the number of grids, it is necessary to perform grid independence verification. In this study, ANSYS-Meshing 2023R1 software is used to mesh three types of serpentine microfluidic chips. Tetrahedral grids were selected for their adaptability to complex geometries. Near-wall regions were refined with three layers of inflation cells to resolve boundary layer effects. The average skewness of the selected mesh was below 0.35, ensuring high quality. Grid independence was confirmed by monitoring the outlet temperature until changes fell below 0.1%. Figure 3 shows the grid independence verification, and Table 3 shows the grid number and convergence analysis. The average fluid outlet temperature is analyzed to determine the influence of the number of grids on the simulation results. It has been observed that the average temperature at the fluid outlet decreases progressively with an increase in the number of grids. When the number of grids is 5.95 million, the average fluid outlet temperature changes less than 0.1%. Considering the accuracy of simulation results and the occupation of computing resources, the fourth grid with 5.95 million grids is finally selected.

**Figure 4:**
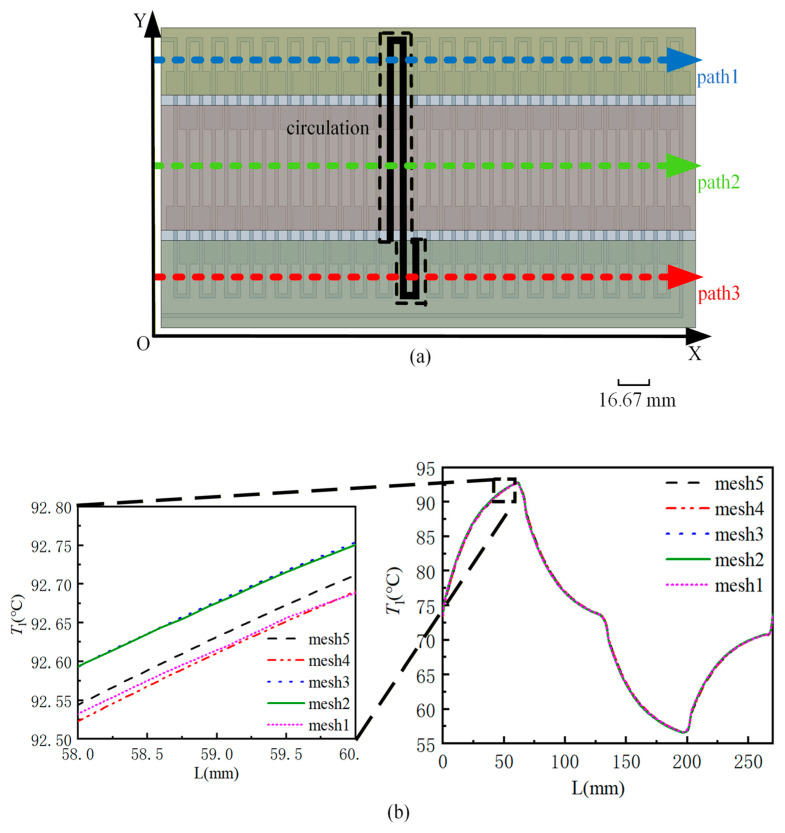
(**a**) Schematic diagram of the circulation taken; (**b**) curve of the fluid temperature on the centerline of different grid numbers with the length of the flow channel.

To facilitate the subsequent analysis of results, this study selects the temperature value on the centerline of a specific circulation channel as the representative temperature of the fluid within that channel. Figure 4a shows the schematic diagram of the circulation taken. In order to avoid the influence of the fluid temperature value on the centerline on the number of grids and the subsequent analysis results, according to the numerical simulation results, the curve of the fluid temperatures on the centerline with the length of the flow channel under different grid numbers are calculated, as shown in Figure 4b. The number of different grids has little influence on the fluid temperature curve, and the temperature difference with the selected fourth grid is within 0.1%. Therefore, the fourth grid with 5.95 million grids can be used to calculate the fluid temperature on the centerline.

## 3. Results and Discussion

### 3.1. Numerical Model Verification

**Figure 5:**
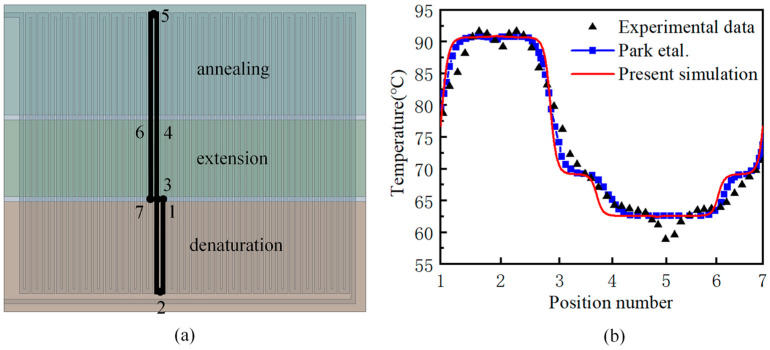
(**a**) Numerical model verification of a PCR device with serpentine channel single-sided integrated heaters; (**b**) comparison of simulation data with experimental data of Park et al. [31,32].

The experimental data of Park et al. [31,32] are used to verify the numerical simulation method. The numerical simulation results of fluid temperature in the flow channel are compared with the experimental results, as shown in Figure 5, The points in Figure 5a correspond to those in Figure 5b, which can verify the correctness of the simulation results in this paper. During the experiment, heat loss occurs due to convection between the experimental device and the surrounding environment, which can lead to deviations between the numerical calculation results and the experimental outcomes. Nevertheless, numerical simulations generally provide a reliable prediction of fluid temperature changes.

### 3.2. Influence of Serpentine Microfluidic Chip Structure on Heat Transfer Characteristics

**Figure 6:**
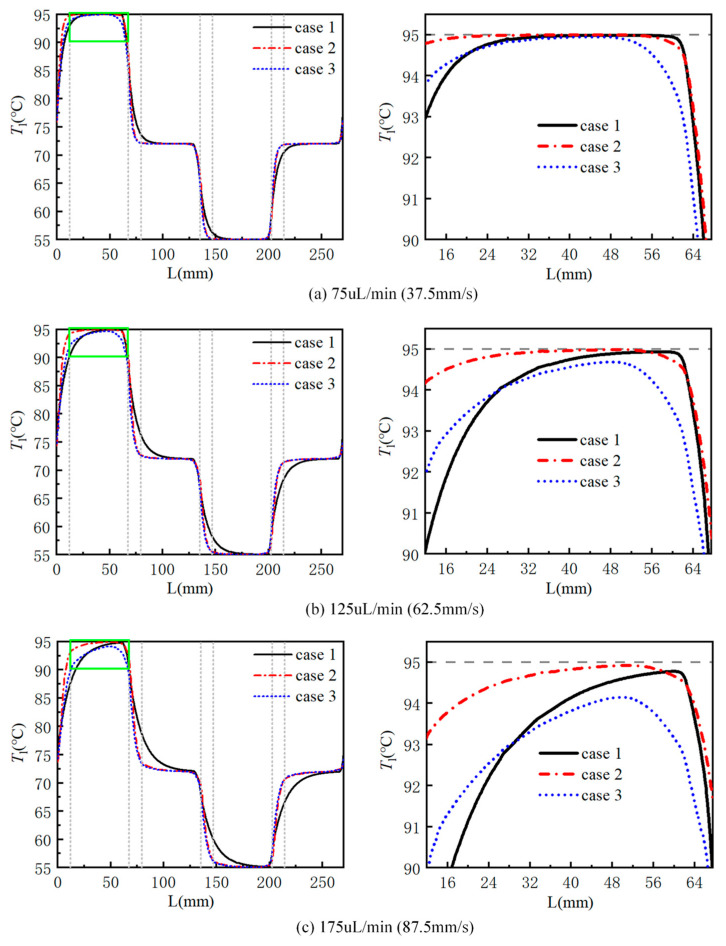
The fluid temperature at the centerline of a cycle varies with the length of the flow channel at different flow rates: (**a**) 75 μL/min; (**b**) 125 μL/min; (**c**) 175 μL/min.

Figure 6 shows the curve of fluid temperature at the centerline with the length of the flow channel in a cycle at flow rates of 75 μL/min (37.5 mm/s), 125 μL/min (62.5 mm/s), and 175 μL/min (87.5 mm/s) for the three structures. The middle part of the two dashed lines in the figure represents the flow channel expansion area, while the outside of the dashed line represents the ordinary straight flow channel area. As can be seen from the figure, under the three flow rates, the transition of the temperature curve of the fluid in case 1 between any two design temperatures is relatively gentle. However, as the flow rate decreases, the temperature curve between any two design temperatures in case 1 gradually becomes steeper. This is because when fluid flows from one temperature zone into another temperature zone, due to large flow rate and fast flow speed, the thermal boundary layer of fluid develops slowly near the inlet of the flow channel. There is a thermal inlet section of fluid flow, which requires a longer distance to stabilize, so the fluid temperature curve is relatively gentle. When the flow rate decreases, the fluid flow speed slows down, the thermal inlet section becomes shorter, and the fluid temperature curve becomes steeper. When the flow rate is 75 μL/min, the maximum heat inlet section in case 1 is 40 mm in the three temperature zones. When the flow rate is 175 μL/min, the maximum heat inlet section in case 1 is 70 mm in three temperature zones. In case 2 and case 3, the fluid temperature curves in the flow channel extension areas are steeper than those in the corresponding positions in case 1. The fluid flow velocity becomes low with the addition of the flow channel expansion areas, which can effectively shorten the length of the hot inlet section. The temperature gradients of the fluid in these areas are larger than those in the ordinary straight flow channel areas.

When the flow rate is 75 μL/min, all three structures successfully meet the design temperature requirements. The fluid temperature in each temperature zone is stable at the design temperature. The effect of the flow channel expansion areas is minimal. When the flow rate increases to 125 μL/min, the fluid velocity increases and the effect of the flow channel expansion areas becomes apparent. In the 95 °C temperature zone, the maximum fluid temperature in case 3 is reduced to 94.7 °C, while the maximum fluid temperature in case 1 and case 2 is 94.9 °C. Among the three structures, case 2 not only has more uniform fluid temperature in each temperature zone and the longest constant temperature area, but also has the best fluid heating effect. When the flow rate continues to increase to 175 μL/min, only case 2 can meet the design temperature requirements. As can be seen from the figure, although the maximum fluid temperature in case 3 is 0.6 °C lower than that in case 1, the temperature curve in the ordinary straight flow channel area within the 95 °C temperature zone is gentler, indicating a more uniform fluid temperature. The constant temperature areas of case 2 and case 3 in the 72 °C and 55 °C temperature zones are 10 mm and 30 mm longer than that of case 1, respectively. In the ordinary straight flow channel areas in the 95 °C temperature zone, the fluid temperature in case 2 is the highest, close to the design temperature. Meanwhile, the fluid temperature curve is the gentlest and the fluid temperature is uniform. This means that at the same flow rate, the length of the flow channel needed to heat to the design temperature is shorter. When the fluid flows from the 95 °C temperature zone to the 72 °C temperature zone in case 2, the fluid temperature in the flow channel extension areas is reduced from 91.7 °C to 72.2 °C, which indicates that the introduction of the flow channel extension areas makes the temperature transition stage between the two temperature zones basically complete in this area.

Improved temperature uniformity ensures that all DNA fragments experience optimal denaturation, annealing, and extension conditions, thereby increasing amplification efficiency and product yield. Conversely, flow dead zones in expansion areas may lead to prolonged residence times, increasing the risk of non-specific binding or primer dimer formation. Therefore, while case 2 improves thermal performance, careful design is needed to minimize dead zones to avoid biochemical inefficiencies.

**Figure 7:**
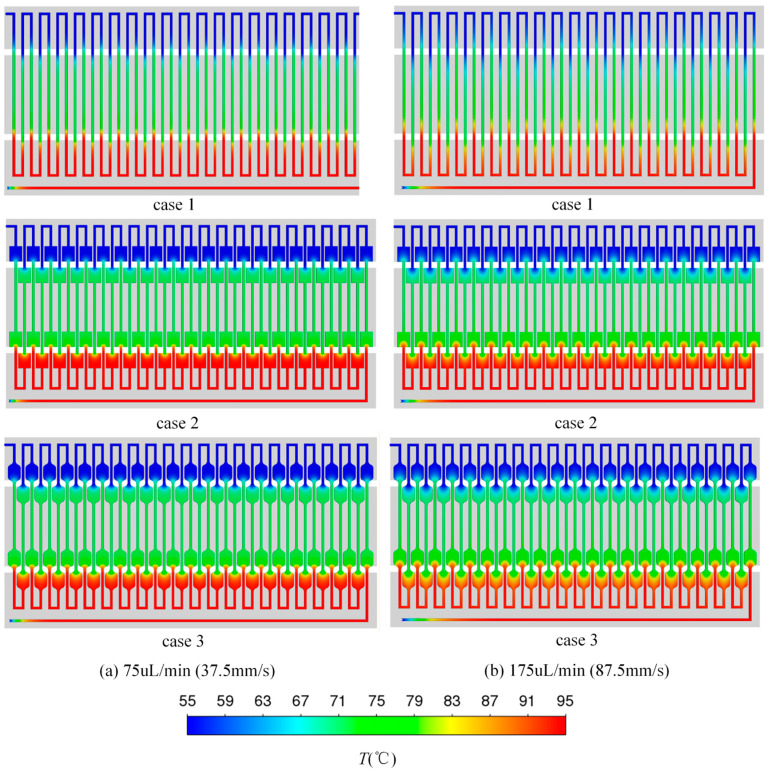
The temperature distribution cloud diagrams of three structures at different flow rates: (**a**) 75 μL/min; (**b**) 175 μL/min.

Figure 7a,b show the temperature contours of three structures at the volume flow rate of 75 μL/min and 175 μL/min, respectively. It can be seen from the temperature contours that under the volume flow rate of 75 μL/min in case 1, the fluid velocity is relatively slow, resulting in a shorter length of the hot inlet section of the flow channel. The fluid temperature in each temperature zone is relatively uniform. However, at the volume flow rate of 175 μL/min, the fluid flow speed is fast. In case 1, when the fluid flows from one temperature zone to another temperature zone, there is a hot inlet section in the fluid flow. This section is relatively long. As a result, the fluid does not reach the design temperature of the first temperature zone before entering the next temperature zone. The fluid temperature difference in the adjacent flow channels is significant, resulting in uneven fluid temperatures within each temperature zone.

To address the issue of the extended length of the hot inlet section at high flow rates, case 2 and case 3 introduce the flow channel expansion areas when the fluid flows into the next temperature zone. Because the flow channel width in the extended areas is larger than in other areas, the flow velocity is relatively low, and the length of the hot inlet section of the flow channel is relatively short. This leads to the fluid temperature in this area approaching the design temperature of the subsequent temperature zone before it flows into the ordinary flow channel. The change in fluid temperature can be basically completed in the expansion areas of the flow channel, and the temperature uniformity of the fluid in the ordinary flow channel can be improved. At the volume flow rate of 75 μL/min, the flow rate is relatively small and the fluid velocity is low. The presence of the flow channel expansion areas in case 2 and case 3 has no obvious effect on improving the temperature uniformity of the fluid in each temperature zone. However, at a volume flow rate of 175 μL/min, the introduction of flow channel expansion areas in cases 2 and case 3 results in longer constant temperature areas compared to case 1. The fluid temperature difference between adjacent flow channels within each temperature zone is reduced, resulting in a more uniform fluid temperature distribution in each temperature zone. However, in comparison to case 2, the chamfering of the flow channel extension areas in case 3 leads to a reduction in the area of these extensions. Consequently, the maximum temperature of the fluid in this region does not reach the maximum temperature observed in case 2.

**Figure 8:**
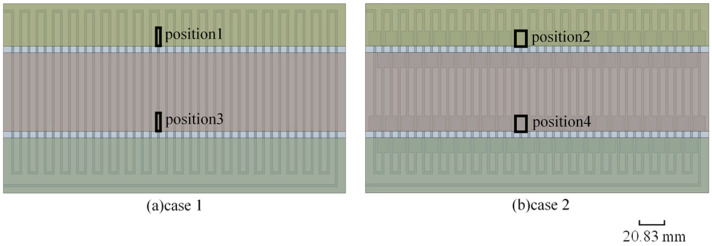
(**a**) The positions of the ordinary straight flow channel area; (**b**) The positions of the flow channel expansion area.

**Figure 9:**
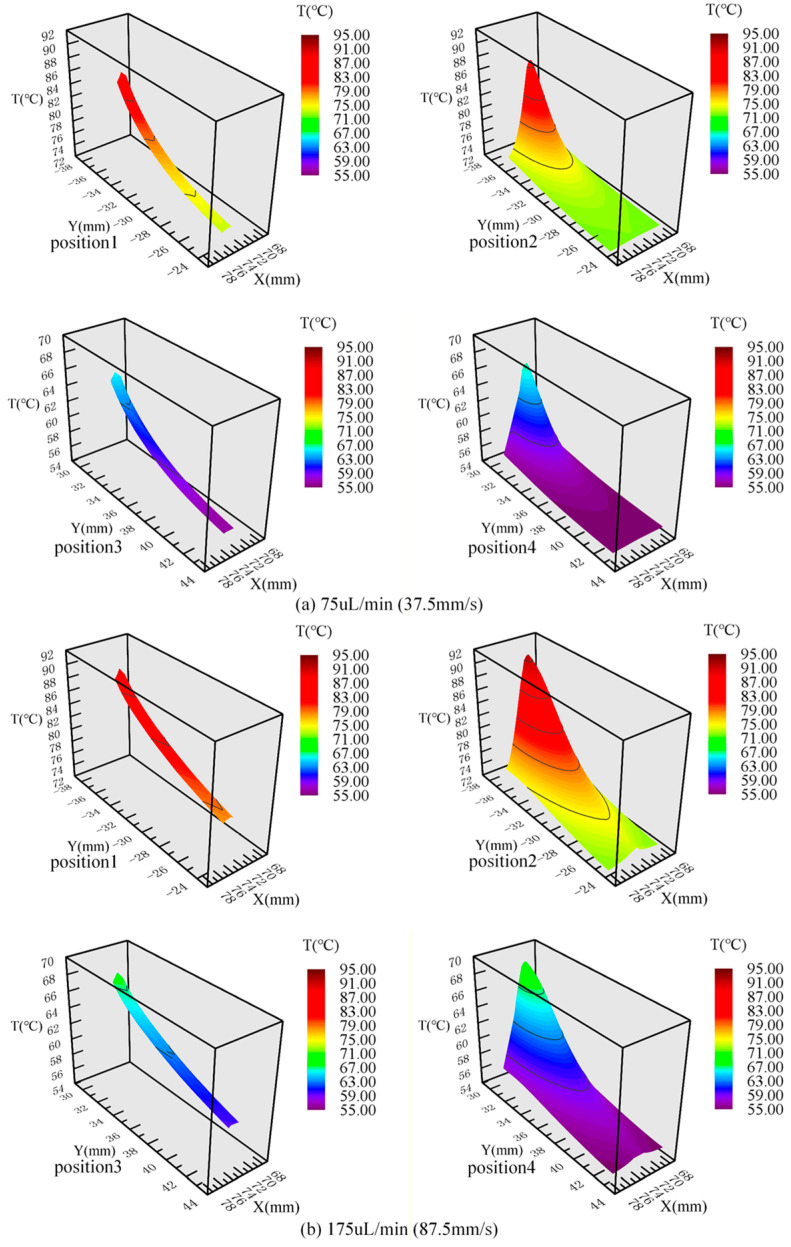
The fluid temperature surface diagrams in the flow channel extension area and the ordinary straight flow channel area at different flow rates: (**a**) 75 μL/min; (**b**) 175 μL/min.

The positions of interest are shown in Figure 8. Figure 9a,b show the fluid temperature in the flow channel extension areas and the corresponding positions in the ordinary straight flow channel areas under the volume flow rate of 75 μL/min and 175 μL/min, respectively. It can be seen from Figure 9 that the fluid temperature gradient in the flow channel extension area is larger than that in the ordinary straight flow channel area at the same position. At the volume flow rate of 75 μL/min, when the fluid flows from the 95 °C temperature zone to the 72 °C temperature zone, the fluid temperature gradient in the ordinary straight flow channel area is 1.08 °C/mm, compared with the fluid temperature gradient in the flow channel expansion area of 1.33 °C/mm. When the fluid flows from the 72 °C temperature zone into the 55 °C temperature zone, the fluid temperature gradient is 0.99 °C/mm in the flow channel extension area, which is about 1.2 times that of the ordinary straight flow channel area at the corresponding position (0.82 °C/mm). At the volume flow rate of 175 μL/min, when the fluid flows from the 95 °C temperature zone to the 72 °C temperature zone, the fluid temperature gradient in the ordinary straight flow channel area is 0.99 °C/mm, compared with the fluid temperature gradient in the flow channel expansion area of 1.63 °C/mm. When the fluid flows from the 72 °C temperature zone into the 55 °C temperature zone, the fluid temperature gradient in the ordinary straight flow channel area at the corresponding position is 0.75 °C/mm, compared with the fluid temperature gradient of 1.21 °C/mm in the flow channel extension area, which is about 1.6 times that of the ordinary straight flow channel area at the corresponding position. Therefore, the introduction of the flow channel extension areas can effectively shorten the length of the inlet section and increase the fluid temperature gradient, so that the fluid temperature can quickly transition to the design temperature of the next temperature zone, and improve the temperature uniformity of the fluid in each temperature zone.

**Figure 10:**
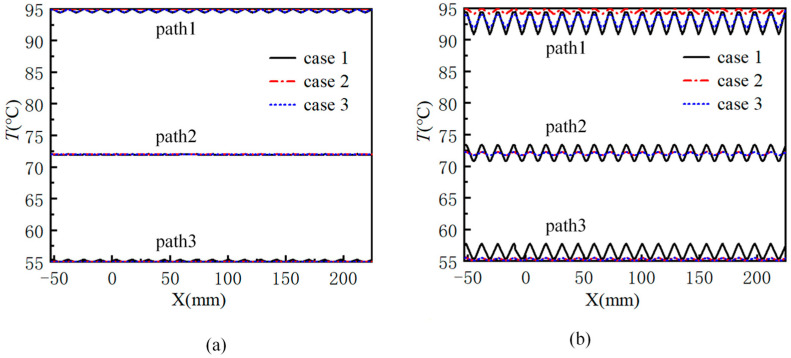
The fluid temperature curves of three structures along different paths at different flow rates: (**a**) 75 μL/min; (**b**) 175 μL/min.

Figure 10 shows the fluid temperature curves of the three structures along different paths at the volume flow rates of 75 μL/min and 175 μL/min. The three paths are path 1, path 2, and path 3 as shown in Figure 4a. At the volume flow rate of 75 μL/min, due to the low flow rate, the introduction of an expanded flow channel has minimal impact on enhancing the temperature uniformity across different temperature zones. It is evident from the figure that the fluid temperature curves of the three structures along paths 2 and 3 closely align. In case 1, the fluid temperature curve along path 1 fluctuates the most, and the maximum temperature difference is 0.4 °C. At the volume flow rate of 175 μL/min, the fluid temperature curve in case 1 fluctuates the most along path 1. The maximum temperature difference is 3.5 °C. In case 2, the fluid temperature curve fluctuates the least along path 1, and the maximum temperature difference in the 95 °C temperature area is only 0.7 °C. The maximum temperature difference is reduced by 80% compared with case 1. The fluid temperature curves of case 2 and case 3 along path 2 and path 3 basically coincide, while the temperature curves along path 1 are quite different. However, the maximum temperature difference of case 3 is 2.1 °C, which is still 40% lower than that of case 1. This fully shows that the introduction of the flow channel expansion areas can help the fluid temperature in the two adjacent ordinary straight flow channels to converge under the condition of large flow, and can improve the temperature uniformity of the fluid in each temperature zone. If the length of the flow channel extension areas is increased appropriately, the fluctuation of the fluid temperature curve under the three paths can be fully reduced, and the temperature uniformity of the fluid in each temperature zone can be improved.

### 3.3. Influence of Serpentine Microfluidic Chip Structure on Velocity Distribution

**Figure 11:**
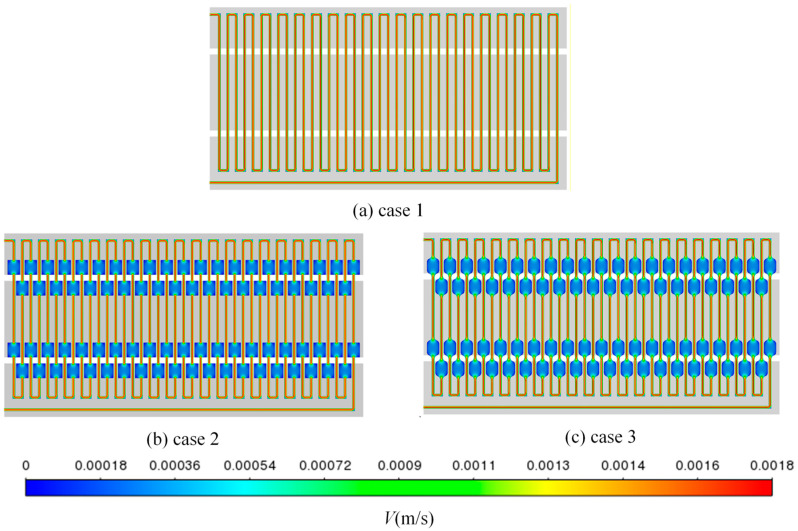
The velocity distribution cloud diagrams of three different structures at a volume flow rate of 175 μL/min.

**Figure 12:**
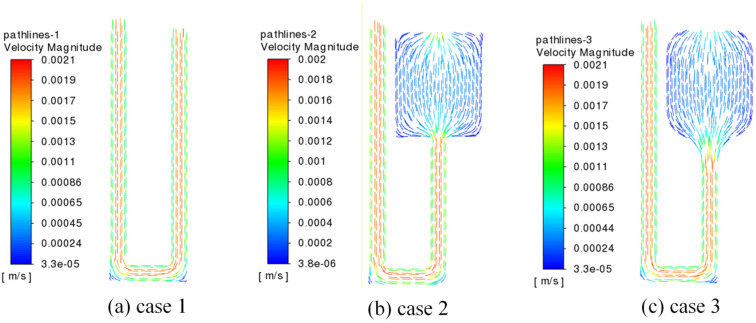
The local path lines diagrams of three structures at a volume flow rate of 175 μL/min.

Figure 11 shows the velocity contours of three different structures at a volume flow rate of 175 μL/min, and Figure 12 shows the local path lines of three structures at a volume flow rate of 175 μL/min. It can be seen that when the flow channel in case 1 is an ordinary straight flow channel, due to the influence of the boundary layer, the flow velocity at the center is significantly higher compared to the slower velocities near the channel walls with non-slip boundaries. At the turn of the flow channel, the flow velocity at the inner bend is relatively high, and the flow velocity at the outer bend is low. This is because the fluid is compressed by the channel wall when turning, and part of the fluid is squeezed to the inner bend of the flow channel, resulting in relatively high flow velocity at the inner bend. At the same time, the fluid will produce a flow dead zone here, resulting in increased flow resistance and pressure drop. The change in fluid velocity in ordinary straight flow channel in case 2 is the same as that in case 1. It is high in the middle of the channel and low around the channel. As the flow channel expansion areas are wider than the ordinary flow channels, the internal flow velocity is relatively low; however, the flow velocity at the four sharp corners in these areas is the lowest. This phenomenon occurs because the fluid flow at these corners is hindered, leading to sudden changes in flow direction and the formation of flow dead zones. In comparison to case 2, the flow channel expansion areas in case 3 are smoother, resulting in a more uniform fluid flow.

### 3.4. Influence of Serpentine Microfluidic Chip Structure on Pressure Drop Characteristics

**Figure 13:**
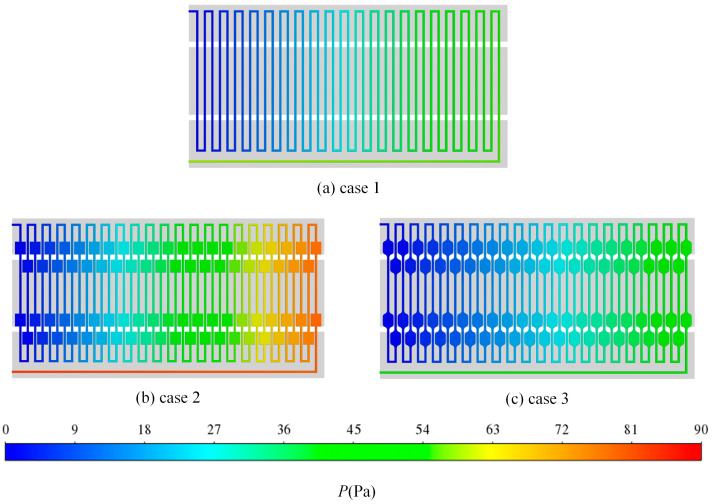
The pressure distribution cloud diagrams of three different structures at a volume flow rate of 175 μL/min.

Figure 13 shows the pressure distribution cloud diagrams of three different structures at a volume flow rate of 175 μL/min. It can be seen from the diagrams that among the three structures, the inlet and outlet pressure drop is larger in case 2, while the inlet and outlet pressure drop is relatively small in case 3. According to the simulation results, the pressure drop at the inlet and outlet in case 1 is 59.03 Pa. In case 2, the pressure drop at the inlet and outlet increases to 83.08 Pa, representing a 41% increase compared to case 1. When the fluid transitions from the ordinary flow channels to the expansion areas, the flow velocity experiences a significant change due to the characteristics of the expansion zone. This abrupt change leads to the formation of flow dead zones at the four sharp corners within this area, resulting in increased flow resistance. Consequently, energy loss occurs, which contributes to a higher pressure drop at both the inlet and outlet. In case 3, the pressure drop at the inlet and outlet is measured at 48.95 Pa, which is 41% lower than that observed in case 2 and 17% lower than in case 1. The chamfering of the flow channel expansion areas effectively reduces the flow dead zones at the sharp corners, thereby decreasing flow resistance. Additionally, the chamfered design facilitates smoother fluid flow within the channels, minimizes friction losses between the fluid and the channel walls, and consequently reduces the pressure drop at both the inlet and outlet. Pressure drop contributions were analyzed: bends accounted for ~30%, expansion zones ~45%, and straight channels ~25% of the total drop in case 2. This indicates that expansion zones are the major source of resistance, suggesting that future designs should optimize these regions to reduce energy loss.

## 4. Conclusions

The purpose of this study is to develop a continuous flow polymerase chain reaction device to ensure the smooth PCR process under the condition of high flow rate. This study compared the differences in fluid temperature distribution, velocity distribution, and pressure loss between two kinds of serpentine double-sided heating microfluidic chips with flow channel expansion areas; ordinary serpentine double-sided heating microfluidic chips were compared by a numerical simulation method, and the influence of flow channel expansion areas on fluid flow and heat transfer characteristics was explored, so as to find the optimal structure under the condition of large flow rate. The main conclusions of this study are as follows:

1. At a flow rate of 75 µL/min, the fluid velocity is slow, and all three structures successfully meet the required design temperature requirements. However, when the flow rate is further increased to 175 µL/min, only case 2 among the three structures can meet the design temperature requirements. Although the maximum fluid temperature in case 3 is 0.6 °C lower than that in case 1, the temperature curve in the ordinary straight flow channel area within the 95 °C temperature zone is gentler, indicating a more uniform fluid temperature. The constant temperature areas of case 2 and case 3 in the 72 °C and 55 °C temperature zones are 10 mm and 30 mm longer than that of case 1, respectively.
2. At a flow rate of 175 µL/min, when the fluid flows from the 72 °C temperature zone into the 55 °C temperature zone, case 2 is about 1.6 times that of the ordinary straight flow channel area at the corresponding position. The introduction of the flow channel extension areas can effectively shorten the length of the inlet section and increase the fluid temperature gradient, so that the fluid temperature can quickly transition to the design temperature of the next temperature zone, and improve the temperature uniformity of the fluid in each temperature zone.
3. At the volume flow rate of 175 μL/min, the fluid temperature curve in case 1 fluctuates the most along path 1. The maximum temperature difference is 3.5 °C. In case 2, the fluid temperature curve fluctuates the least along path 1, and the maximum temperature difference in the 95 °C temperature area is only 0.7 °C. The maximum temperature difference is reduced by 80% compared with case 1.
4. As the flow channel expansion areas are wider than the ordinary flow channels, the internal flow velocity is relatively low; however, the flow velocity at the four sharp corners in these areas is the lowest. This phenomenon occurs because the fluid flow at these corners is hindered, leading to sudden changes in flow direction and the formation of flow dead zones. Consequently, energy loss occurs, which contributes to a higher pressure drop at both the inlet and outlet. In case 2, the pressure drop at the inlet and outlet increases to 83.08 Pa, representing a 41% increase compared to case 1. When considering the overall performance, the serpentine double-sided heating microfluidic chip with unchamfered flow channel expansion areas (case 2) represents the optimal structure.
5. This study is primarily based on numerical simulations, and the predicted temperature and pressure characteristics require further confirmation through experimental testing. Additionally, there remains room for optimizing the geometric shape of the flow channel expansion zones to enhance heat transfer performance while simultaneously reducing pressure losses. Future work could also explore the influence of different fluid media or surface modifications on PCR chip performance.

## Figures and Tables

**Table 1:** Geometric parameters of three structures of serpentine microfluidic chips.

Structure	Parameter	Variable	Value
Case 1	chip length	l_t_	293.48 mm
	chip width	w_t_	150.06 mm
	denaturation zone width	w_d_	43.78 mm
	extension zone width	w_e_	62.5 mm
	annealing zone width	w_a_	33.78 mm
	channel width	w_c_	2 mm
	channel depth	h_c_	1 mm
	thickness of heating blocks	h_1_/h_3_	1 mm
	chip thickness	h_t_	3 mm
Case 2/Case 3	chip length	l_t_	293.48 mm
	chip width	w_t_	150.06 mm
	denaturation zone width	w_d_	43.78 mm
	extension zone width	w_e_	62.5 mm
	annealing zone width	w_a_	33.78 mm
	channel width	w_c_	2 mm
	channel depth	h_c_	1 mm
	thickness of heating blocks	h_1_/h_3_	1 mm
	chip thickness	h_t_	3 mm
	length of flow channel extension area	l_ex_	12 mm
	width of flow channel extension area	w_ex_	10 mm

**Table 2:** Physical property parameters of materials used in numerical simulation.

	Copper	PMMA	Water
ρ [kg/m^3^]	8978	1170	998.2
μ [kg/(m·s)]			0.001003
c_p_ [J/(kg·K)]	381	1470	4182
k [W/(m·K)]	387.6	0.17	0.6

**Table 3:** Grid number and convergence analysis.

Mesh	Number (Million)	T_out_ (℃)	Relative Error
Mesh 1	1.24	59.7828	0.51%
Mesh 2	2.47	59.57613	0.16%
Mesh 3	3.62	59.55042	0.12%
Mesh 4	5.95	59.52665	0.08%
Mesh 5	7.82	59.47818	-

## Data Availability

The original contributions presented in the study are included in the article, further inquiries can be directed to the corresponding author.

## Abbreviations

The following abbreviations are used in this manuscript:

PCR	Polymerase chain reaction
PMMA	Polymethyl Methacrylate
